# Uncoupling protein 1 knockout aggravates isoproterenol-induced acute myocardial ischemia via AMPK/mTOR/PPARα pathways in rats

**DOI:** 10.1007/s11248-021-00289-0

**Published:** 2021-10-28

**Authors:** Daorong Hou, Heling Fu, Yuan Zheng, Dan Lu, Yuanwu Ma, Yuan Yin, Lianfeng Zhang, Dan Bao

**Affiliations:** 1 Key laboratory of the model animal, Animal Core Facility of Nanjing Medical University, 101 Longmian Avenue, Nanjing, 211166 China; 2 Key laboratory of human disease comparative medicine, NHFPC, Institute of Laboratory Animal Science, Chinese Academy of Medical Sciences and Comparative Medical Center, Peking Union Medical College, Beijing, 100021 China

**Keywords:** UCP1, Acute myocardial ischemia, LV remodeling, Oxidative stress

## Abstract

Uncoupling protein 1 (UCP1) was found exclusively in the inner membranes of the mitochondria of brown adipose tissue (BAT). We found that UCP1 was also expressed in heart tissue and significantly upregulated in isoproterenol (ISO)-induced acute myocardial ischemia (AMI) rat model. The present study is to determine the underlying mechanism involved in the UCP1 upregulation in ISO-induced AMI rat model. The *Ucp1*^*−/−*^ rats were generated by CRISPR-Cas9 system and presented decreased BAT volume. 2-months old Sprague Dawley (SD) wild-type (WT) and *Ucp1*^*−/−*^ rats were treated with ISO intraperitoneally 30 mg/kg once a day for 3 consecutive days to establish AMI model. In saline group, the echocardiographic parameters, serum markers of myocardial injury cardiac troponin I (cTnI), creatine kinase isoenzyme MB (CK-MB), oxidant malondialdehyde (MDA), antioxidant superoxide dismutase (SOD) or fibrosis were comparable between WT and *Ucp1*^*−/−*^ rats. ISO treatment induced worse left ventricle (LV) hypertrophy, myocardial fibrosis, increased higher cTnI, CK-MB and MDA and decreased lower SOD level in *Ucp1*^*−/−*^ rats compared with that of WT rats. *Ucp1*^*−/−*^ rats also presented lower myocardial phosphocreatine (PCr)/ATP-ratio, which demonstrated worse cardiac energy regulation defect. ISO treatment induced the phosphorylation of AMP-activated protein kinase (AMPK) activation, subsequently the phosphorylation of mammalian target of rapamycin (mTOR) inhibition and peroxisome proliferators-activated receptor α (PPARα) activation in WT rats, whereas activation of AMPK/mTOR/PPARα pathways significantly inhibited in *Ucp1*^*−/−*^ rats. To sum up, UCP1 knockout aggravated ISO-induced AMI by inhibiting AMPK/mTOR/PPARα pathways in rats. Increasing UCP1 expression in heart tissue may be a cytoprotective therapeutic strategy for AMI.

**Supplementary Information:**

The online version contains supplementary material available at 10.1007/s11248-021-00289-0.

## Introduction

Acute myocardial ischemia (AMI) is a leading cause of acute myocardial infarction which with high mortality (7%) and morbidity (22%) worldwide and is characterized by an acute condition of myocardial ischemic necrosis caused by the interruption in the supply of myocardial oxygen and nutrients (Meng et al. 2018). During ischemia, intracellular energy metabolism is abnormal and oxidative stress increases with large reactive oxygen species (ROS) generation (Vanden Hoek et al. 1997). ROS are generally considered as toxic byproducts of aerobic metabolism and the primary cause of cell damage. It is well established that mitochondria are a main source of cellular ROS(Murphy, 2009) and mitochondrial ROS production is steeply dependent on electrochemical proton gradient (△*p*) (Cadenas, 2018) which drives adenosine triphosphate (ATP) synthesis by F_0_F_1_-ATPase, correspondingly, control mitochondrial ROS generation has been identified as a cytoprotective strategy under conditions of oxidative stress.

Uncoupling proteins (UCPs) are members of the anion carrier proteins superfamily which exist in the inner membrane of mitochondria and able to induce proton leak. Proton leak increases the respiration rate, dissipates the △*p*, induces partial mitochondrial oxidative phosphorylation uncoupling and generates heat instead of ATP, thus diminishing mitochondrial ROS production (Akhmedov et al. 2015; Sack 2006).

The transcriptional regulation of the UCP genes was mainly mediated by peroxisome proliferators-activated receptor (PPAR) subtypes, with a distinct relevance depending on the UCP gene and the tissue in which it is expressed (Villarroya et al. 2007). UCP1, the first described uncoupling protein, is expressed largely in brown adipose tissue (BAT) of mammals (Meyer et al. 2010). Since PPARα is preferentially expressed in BAT, *Ucp1* gene is induced mainly through PPARα, which is a downstream signaling molecule of AMP-activated protein kinase (AMPK) / mammalian target of rapamycin (mTOR) pathways. UCP1 allows the influx of protons from the mitochondrial intermembrane space to the matrix thereby uncoupling mitochondrial respiration from ATP-synthesis. When UCP1 or BAT is activated, the brown adipocytes consume glucose and lipids, converting the energy of oxidation of fatty acids and glucose into heat (Cannon and Nedergaard 2004). The unique metabolic and thermogenic properties of UCP1 and BAT have raised concerns about their potential therapeutic applications for metabolic diseases, such as obesity and type II diabetes (Townsend and Tseng 2012; Tseng et al. 2010).

Isoproterenol (ISO), a synthetic catecholamine and β-adrenergic receptor agonist, produces severe myocardial stress when administered in supramaximal dose. It is also documented that the pathophysiological and morphological alterations induced by ISO in the heart tissues of experimental animals are similar to those observed in human myocardial infarction (Nagoor Meeran et al. 2012; Patel et al. 2010). In the present study, we found that UCP1 is also expressed in heart tissue and the expression of UCP1 is significantly upregulated in ISO-induced AMI (Li et al. 2016; Yang et al. 2011) rat model, suggesting that UCP1 could be involved in the pathogenesis of AMI. Current studies indicate that overexpression of UCP1 resists hypoxia/reoxygenation injury in H9C2 cell (Bienengraeber et al. 2003); overexpression of UCP1 protects against ischemic-reperfusion damage in transgenic mice (Hoerter et al. 2004). However, the potential mechanism of UCP1 under ischemic stress conditions has not yet been investigated. Thus, the aims of the present study are to determine the underlying mechanism involved in the UCP1 upregulation in ISO-induced AMI rat model and provide a strategy for control the development of AMI.

## Materials and methods

### Animals

The *Ucp1* knockout rats were generated by Clustered Regularly Interspaced Short Palindromic Repeats (CRISPR)/CRISPR-associated (Cas) 9 system. In brief, targeting on the exon 1 of *Ucp1*, we designed three pairs of synthesized oligonucleotides gRNA (gRNA sequences were shown in supplementary material Table S1), which were annealed and cloned into the pUC57-gRNA expression plasmids (Plasmid #51,132, Addgene, USA). Both the gRNA expression plasmids and the Cas9 expression plasmid (Plasmid #44,758, Addgene, USA) were linearized and used as templates for in vitro transcription (MEGAshortscript Kit, AM1354, Ambion, USA; T7 Ultra Kit, AM1345, Ambion, USA). A mixture of purified and transcribed Cas9 mRNA and gRNA was microinjected into SD rat zygotes to generate the *Ucp1*^*−/−*^ rat. We performed PCR to identify the genotype of rats and primers were shown in supplementary material Table S2. For genotyping, fragment of WT was 594-bp and fragment of the *Ucp1* knockout gene was 362-bp. There were 30 PCR cycles consisting of 94 °C for 30 s, 65 °C for 30 s and 72 °C for 45 s.

All rats used in the present study were bred in barrier facility (SYXK(Su)2020–0022) and were provided with standard food and water ad libitum. The room temperature kept at 23 ± 2 °C with a 12:12 h light/dark cycle. The ratio of genders was approximately 1:1.

### ISO-induced AMI rat model

At 2 months of age, ISO (I6504, Sigma-Aldrich, USA) was administered intraperitoneally 30 mg/kg once a day for 3 consecutive days in both WT and *Ucp1*^*−/−*^ rats, while the control group was injected with saline. Rats were randomly assigned to WT-saline, *Ucp1*^*−/−*^-saline, WT-ISO or *Ucp1*^*−/−*^-ISO groups (*n* = 20/group). After the saline or ISO administration, the four groups of rats mentioned above were performed electrocardiograph (ECG) examination using BIOPAC MP150 physiological signal acquisition system. Briefly, rats were anesthetized with 1.5% isoflurane and placed in supine position. The ECG electrodes were fixed on both upper and right lower limbs of rats and the standard II-lead ECG was recorded. The S-T segment alteration and/or T wave inversion of ECG indicated the successful establishment of ISO-induced AMI models (supplementary material Figure S1).

### Echocardiography

Rats were anesthetized with 1.5% isoflurane (Table Top Anesthesia Machine Model V-1, Vet Equip Inc., USA) and in vivo cardiac geometry and function was assessed, in the double-blind method, by transthoracic 2D/M-mode echocardiography (MS250 transducer, Vevo 2100, Visual Sonics, Canada).

### Histological analysis

Rats were euthanized with carbon dioxide (CO_2_) inhalation. In brief, we put rats into CO_2_ chamber and turn on CO_2_. The CO_2_ flow rate must displace no more than 30% of the chamber volume per minute. After 2 min, we turned off CO_2_, waited for 5 min and meanwhile observed the physiology of rats. After the rats were confirmed dead, the hearts were removed. For light microscopy, heart tissues were fixed in 4% formaldehyde, mounted in paraffin blocks, then cut into 4 μm thick sections and stained with hematoxylin and eosin (H&E) staining (C0105, Beyotime, China). Collagen content in cardiac tissue sections was stained using Masson Trichrome Stain Kit (HT15, Sigma-Aldrich, USA). Myocardial staining was analyzed by the Pannoramic Viewer 1.15.3 software. For electron microscopy, heart tissues were fixed in 2.5% glutaraldehyde in 0.1 M phosphate buffer (pH 7.4) and postfixed in 1% osmium tetroxide buffer for 1 h. The sections were stained with uranyl acetate and lead citrate and examined under a transmission electron microscope (TEM, JEM-1010, JEOL Ltd, Japan).

### Survival analysis

The cumulative percent mortality was calculated every day for ISO-treated rats. Upon death, rats underwent autopsy, and the pathological changes in the heart were recorded. Kaplan–Meier curves were assayed using log-rank test (SPSS version 16.0 software).

### Biochemical analysis

Rats were anesthetized with 4% isoflurane (Table Top Anesthesia Machine Model V-1, Vet Equip Inc., USA). The whole blood was collected from abdominal aorta. The collected whole blood was placed at room temperature for 30 min and centrifuged at 3000 g for 10 min at 4 °C to harvest the serum. For cardiac enzymology assays, the serum concentrations of lactate dehydrogenase (LDH, 990–63,193, Wako, Japan) and creatine kinase isoenzyme MB (CK-MB, H007T, Medicalsystem Biotechnology, China) were detected using an automatic biochemistry analyzer (HITACHI 7100, Japan). Cardiac troponin I (cTnI) was measured using commercially available kit (LP-R03712, LanpaiBio, China). For oxidative stress parameters, the serum levels of malondialdehyde (MDA), glutathione (GSH) and total antioxidant capacity (T-AOC) were spectrophotometrically measured using diagnostic kits respectively (A003-1, A006-2, A015-2, Jiancheng, China) according to the manufacture’s instruction. Serum superoxide dismutase (SOD, H439, Medicalsystem Biotechnology, China) was measured using an automatic biochemistry analyzer (7100, HITACHI, Japan).

### In situ TUNEL staining assay

A terminal deoxynucleotidyl transferase- (TdT-) mediated deoxyuridine triphosphate (dUTP) nick end labeling (TUNEL) assay was performed according to the manufacturer’s instructions (11,684,817,910, Roche, Switzerland). Heart tissues were fixed in 4% paraformaldehyde overnight, dehydrated, embedded in paraffin, sectioned into 4 μm thick sections, and placed on numbered polylysine-coated glass slides. The deparaffinized tissue sections were incubated with proteinase K (20 mg/ml, Sigma-Aldrich, USA) in a humidified chamber for 15 min, and endogenous peroxidase activity was blocked by treating the sections with 3% H2O2 for 10 min. The sections were then incubated with TdT labeling buffer at 37° C for 1 h in a moist chamber and then counterstained with DAPI. TUNEL-positive cells were stained green, and nuclei were stained blue.

### Magnetic resonance (MR) imaging

All the MR procedures were performed on a horizontal 7.0 T system (Biospec 7 T/20 USR, Bruker, Germany) and used a rat body coil and a tunable ^1^H/^31^P 20 mm surface coil for transmission and reception. Rats were anesthetized with 1.5% isoflurane (Table Top Anesthesia Machine Model V-1, Vet Equip Inc., USA). We used a monitoring system (Small Animal Instruments Inc., Stony Brook, USA) to monitor the heart rate and respiratory rate of rats continuously. T1_MSME (multi-layer multi-echo) was used for the anatomical imaging of BAT. Acquisition parameters were: 55 × 45 mm field of view (FOV), 128 × 128 matrix size, 1 mm slice thickness, echo time (TE) 15 ms, repetition time (TR) 500 ms, 3 averages, 90° flip angle. ^1^H MR-imaging was used for the cardiac anatomical location. Acquisition parameters were: 50 × 50 mm FOV, 256 × 256 matrix size, 1 mm slice thickness, TE 3.5 ms, TR 125 ms, 2 averages, 65° flip angle. ^31^P MR-spectroscopy was used for myocardial phosphocreatine (PCr) and ATP detection to assess myocardium energetics. Acquisition parameters were: 22.8° flip angle, TR 1.5 s, 6 kHz bandwidth, 2048 points, 128 averages. BAT volume was quantitatively analyzed with ImageJ software. ^31^P MR-spectroscopy was analyzed with Mest Re Nova 14.0 software.

### Western blot

The protein concentration of ventricular extracts was measured using the bicinchoninic acid assay (BCA1, Sigma-Aldrich, USA). 30 μg of total extract protein from rat ventricular extracts were separated by SDS-PAGE and then wet transferred onto nitrocellulose membranes (HATF00010, Millipore, USA). Membranes were immunoblotted with primary antibodies against UCP1 (ab10983, Abcam, USA), AMPK α1 + AMPK α2 (ab80039, Abcam, USA), phosphor-AMPK α1 (T183) + AMPK α2 (T172) (ab133448, Abcam, USA), mTOR (2972S, Cell Signaling Technology, USA) and phosphor-mTOR (S2448) (2971, Cell Signaling Technology, USA), PPARα (ab24509, Abcam, USA) and phosphor-PPARα (S12) (ab3484, Abcam, USA). Protein bands were detected using horseradish peroxidase-conjugated secondary antibodies using chemiluminescent detection reagent (sc-2048, Santa Cruz, USA). GAPDH (ab9482, Abcam, USA) was used as normalization. Bands were quantitatively analyzed with ImageJ software.

### Statistical analysis

Normal distribution of the sample sets was determined by Shapiro–Wilk normality test. For sample sets with gaussian distribution, Student 2-tailed *t* test or 2-way ANOVA was used. For the sample sets with a non-gaussian distribution, Mann–Whitney test or Kruskal–Wallis test was used. *P* values < 0.05 were considered significant. Statistical analysis was performed using Prism software (GraphPad).

## Results

### UCP1 upregulated in heart tissue of ISO-induced AMI rat model

**Fig. 1:**
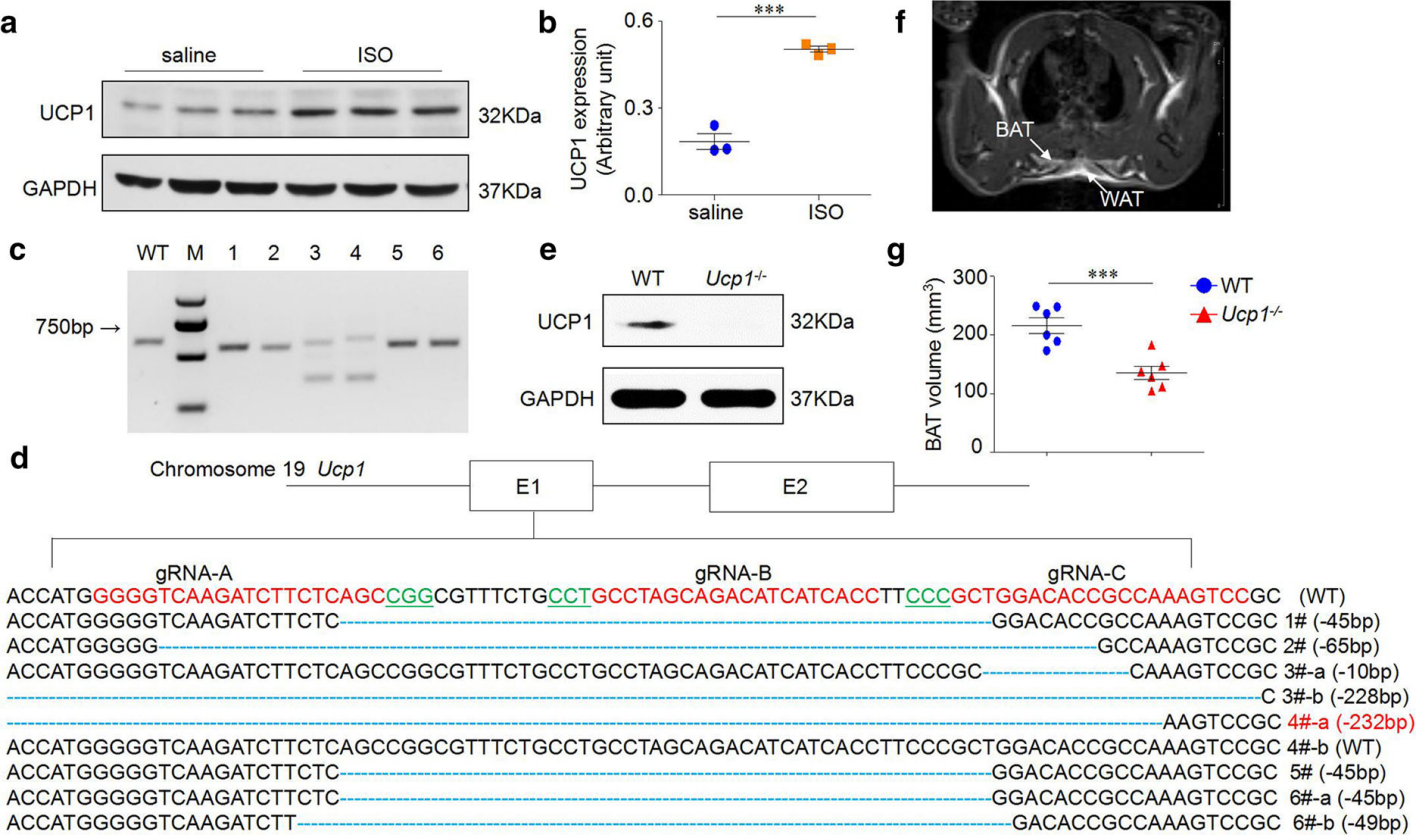
Upregulation of UCP1 in ISO-induced AMI rat model and the generation of *Ucp1* knockout rat. **a** UCP1 expression in heart tissues of ISO-induced AMI rat model. **b** Quantitative analysis of UCP1 expression using GAPDH for normalization (*n* = 3/group, Student’s t-test, ***P < 0.001 *versus* saline group). **c** Target loci of *Ucp1* were amplified using genomic DNA templates from founders. WT: Template DNA was replaced with wild-type genomic DNA; M: DNA molecular weight marker DL2000; 1–6: Founder rats generated by microinjection. **d** PCR products of the targeted fragment in the *Ucp1* in rats were sequenced. The protospacer adjacent motif (PAM) sequence was underlined and highlighted in green; the targeting sites red; deletions (-) were shown to the right of each allele. The E1 and E2 represents exon 1 and exon 2 of *Ucp1* respectively. **e** Protein level of UCP1 in the heart tissues of WT littermates and *Ucp1*^*−/−*^ rats were detected by western blot, using GAPDH as normalization. **f** A typical T1_MSME image allowing BAT to be clearly differentiated from surrounding WAT under physiological conditions. **g** BAT volume in WT and *Ucp1*^*−/−*^ rats at 1 month of age (*n* = 6/group, Student’s t-test, ***P < 0.001 *versus* WT littermates)

We analyzed the expression of UCP1 in heart tissues from WT rats treated with saline and ISO intraperitoneal injection, respectively, and the results indicated that UCP1 expression was increased significantly by 1.7-fold in heart tissues from ISO-induced AMI rat model compared with saline controls (*n* = 3 in the saline and ISO group, respectively, *P* < 0.001, Fig. 1a, b).

### Generation of *Ucp1* knockout rat using CRISPR/Cas9 system

We generated *Ucp1* knockout rat using CRISPR/Cas9 system. The PCR amplification (Fig. 1c) and further sequencing analysis (Fig. 1d) were both performed. The results demonstrated that there were six rats (founder 1–6) had a frame shift mutation. We chose Founder 4, which carried a 232-bp deletion from No.62 bp to 293 bp in the *Ucp1* genome DNA sequence (NC_005118), to establish a colony (designated as *Ucp1*^*−/−*^). We also detected the expression of UCP1 in the heart tissue of the *Ucp1*^*−/−*^ rats by Western blot analysis and the result confirmed the absence of UCP1 in the heart tissue (Fig. 1e).

### UCP1 knockout decreased BAT volume at baseline

UCP1 is expressed largely in BAT of mammals. To determine whether knockout of UCP1 expression could affect BAT, we performed BAT anatomical imaging with T1_MSME MR imaging at 1 month of age. The results showed that BAT was located posterior to white adipose tissue (WAT) in the shoulder blade of rats, which was characterized by bi-lobed shape and signal hypo-intensity relative to overlying WAT (Fig. 1f). Our results also demonstrated that BAT volume was significantly decreased in *Ucp1*^*−/−*^ rats compared with that of WT littermates (135.55 ± 28.30 mm^3^ versus 216.16 ± 32.39 mm^3^, *P* < 0.001, Fig. 1g), suggesting that UCP1 knockout would affect BAT volume.

### UCP1 knockout aggravated adverse left ventricle remodeling in ISO-induced AMI rat model

**Fig. 2:**
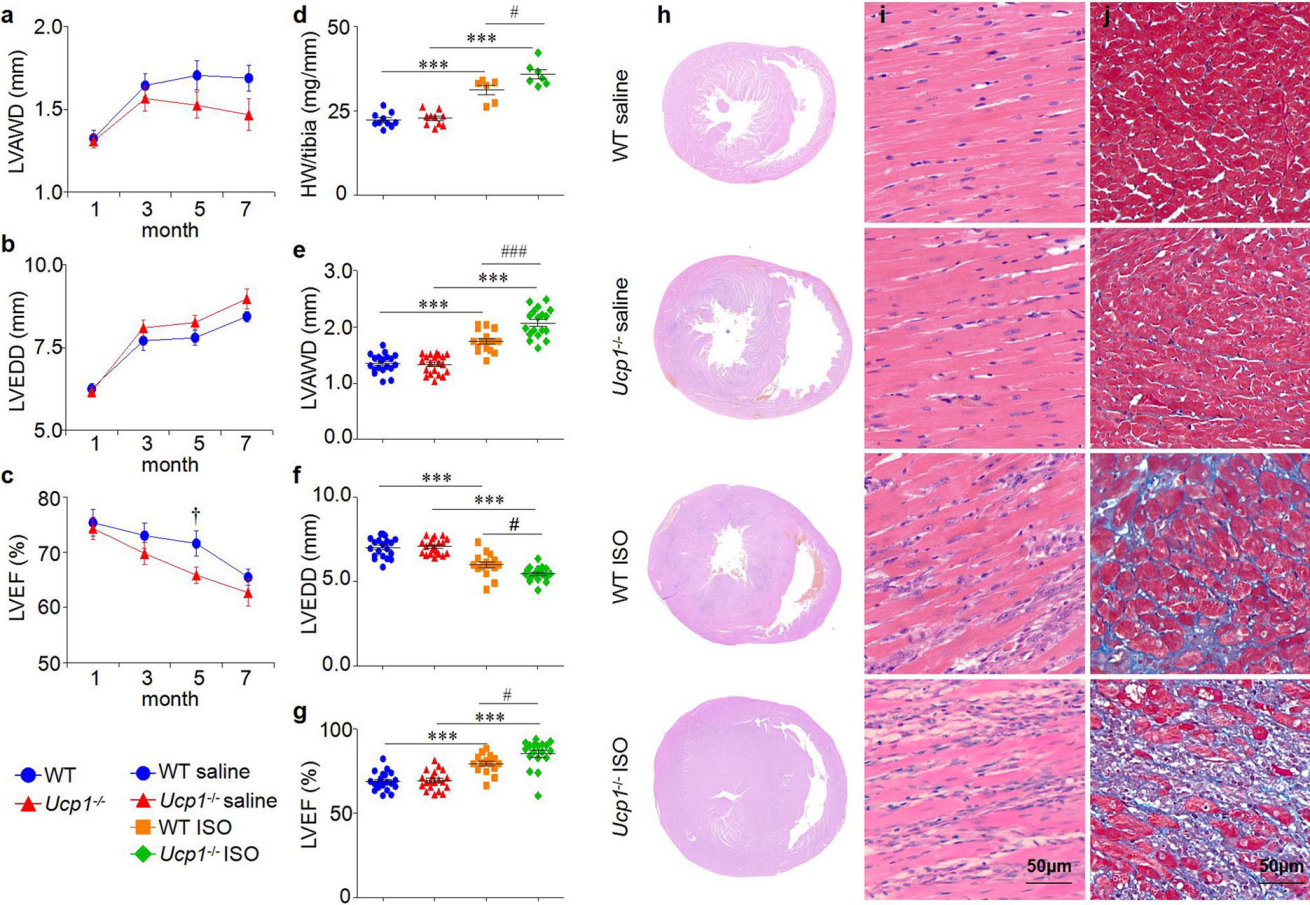
UCP1 knockout aggravated adverse LV remodeling in ISO-induced AMI rat model. **a**–**c** Echocardiographic parameters of LVAWD, LVEDD and LVEF were analyzed in WT and *Ucp1*^−/−^ rats at 1, 3, 5 and 7 months of age (*n* = 17 and 16, 18 and 20, 15 and 16, 15 and 16, respectively, in WT and *Ucp1*^−/−^ rats at 1, 3, 5 and 7 months of age, Student’s t-test, †*P* < 0.05*versus* WT rats). **d** Heart weight to tibia length (HW/tibia) ratio in WT and *Ucp1*^−/−^ rats with saline or ISO treatment after 3 days (*n* = 10 in the WT saline group, *n* = 10 in the *Ucp1*^−/−^ saline group, *n* = 6 in the WT ISO group, *n* = 7 in *Ucp1*^−/−^ ISO group, one-way ANOVA, ***P < 0.001 *versus* saline treatment of the same strain,^#^*P* < 0.05 *versus* ISO treatment in WT rats). **e**–**g** Echocardiographic parameters of LVAWD, LVEDD and LVEF were analyzed in WT and *Ucp1*^−/−^ rats with saline or ISO treatment after 3 days (WT saline group *n* = 20, *Ucp1*^−/−^ saline group *n* = 20, WT ISO group *n* = 17, *Ucp1*^−/−^ ISO group *n* = 18, one-way ANOVA, ***P < 0.001 *versus* saline treatment of the same strain,^#^*P* < 0.05,^###^*P* < 0.001 *versus* ISO treatment in WT rats). **h** H&E staining of the whole-heart transverse sections after ISO treatment. **i**–**j** Magnification of H&E-stained and Masson trichrome-stained LV section (magnification × 400, scan bar is 50 μm). Myocytes stained red and collagen stained blue

To verify whether knockout of UCP1 expression could affect the cardiac morphology and function in AMI rat model induced by ISO, firstly, we performed M-mode echocardiography at 1, 3, 5 and 7 months of age on *Ucp1*^−/−^ rats as well as WT littermates, and the results indicated that *Ucp1*^−/−^ rats presented larger left ventricular diameters, thin-walled ventricles and lower cardiac systolic function compared with that of WT littermates at 5 months of age (Fig. 2a–c and supplementary material Table S3).

Furthermore, we established ISO-induced AMI model in both WT and *Ucp1*^*−/−*^ rats at 2 months of age. Survival analysis, M-mode echocardiography and histological examination were detected. There was no significant difference of survival percent between WT and *Ucp1*^*−/−*^ rats after ISO treatment (supplementary material Figure S2). ISO treatment for 3 days induced left ventricle (LV) hypertrophy (increases in LV mass and wall thickness, decreases in LV diameter) in both WT and *Ucp1*^*−/−*^ rats. However, the degree of hypertrophy induced by ISO was greater in *Ucp1*^*−/−*^ rats than in WT littermates ((LV mass which was evaluated by the index of heart weight and tibia length ratio increased by 56.48% *versus* 39.25%, *P* < 0.05; LV anterior wall at end-diastole (LVAWD) increased by 54.99% *versus* 28.98%, *P* < 0.001; LV end-diastole diameter (LVEDD) decreased by 22.80% *versus* 14.55%, *P* < 0.05, Fig. 2d–f and supplementary material Table S4)). ISO treatment also induced higher compensatory LV systolic function in *Ucp1*^*−/−*^ rats than in WT littermates (LV ejection fraction (LVEF) 85.46% ± 8.35% *versus* 79.47% ± 5.38%, *P* < 0.05, Fig. 2G), while LV systolic function dropped more markedly (LVEF 53.78% ± 2.48% *versus* 58.54% ± 3.10%, *P* < 0.05, supplementary material Table S5 and Figure S3) at 1 month after ISO treatment in *Ucp1*^*−/−*^ rats. Moreover, UCP1 knockout increased myocardial edema with lots of inflammatory cells and fibrosis induced by ISO in *Ucp1*^*−/−*^ rats compared with WT littermates (Fig. 2h–j). Thus, UCP1 knockout predisposed the myocardium to a greater degree of adverse remodeling.

### UCP1 knockout increased myocardial injury, oxidative stress, apoptosis and mitochondria injury in ISO-induced AMI rat model

**Fig. 3:**
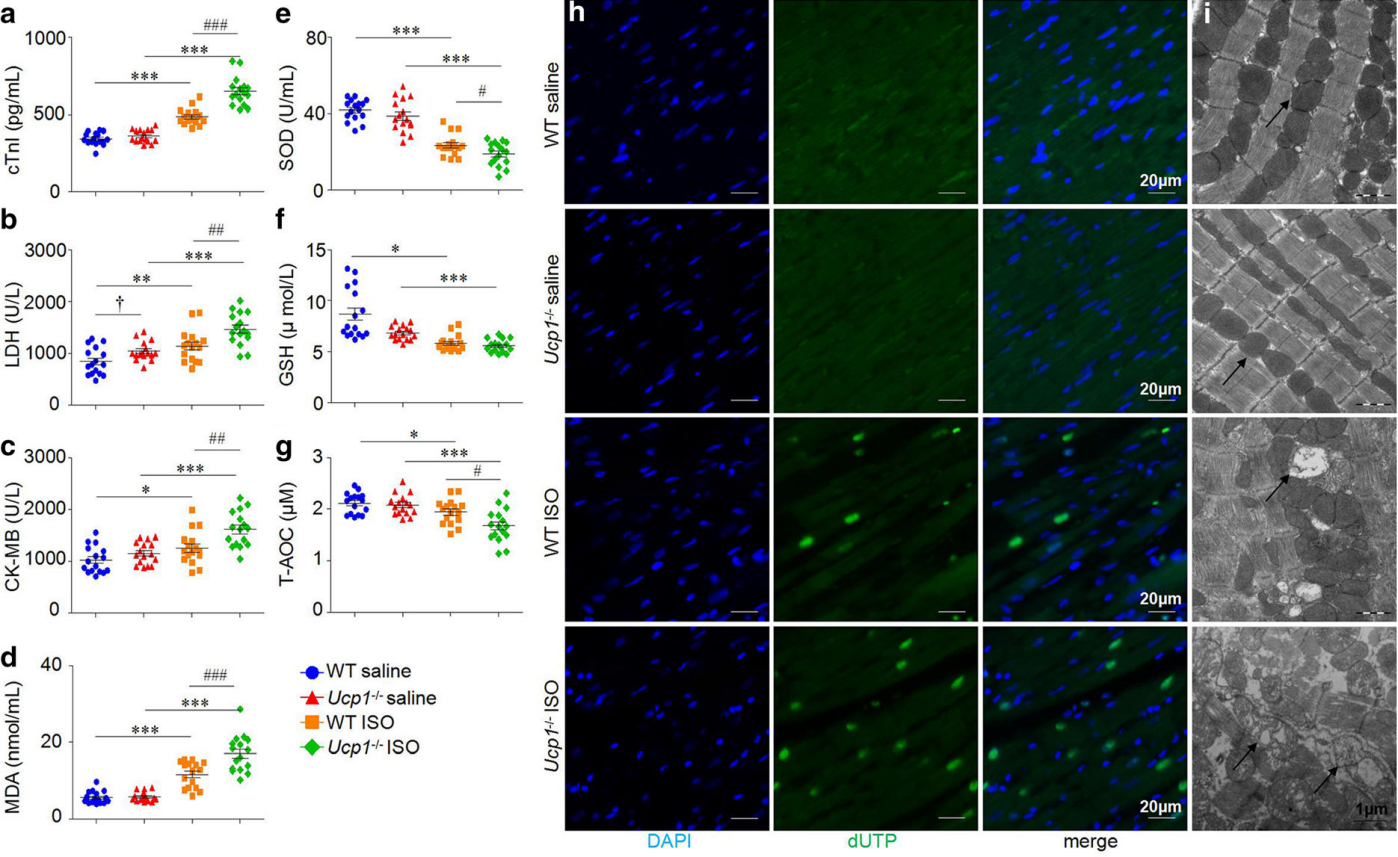
UCP1 knockout increased myocardial injury, oxidative stress and apoptosis in ISO-induced AMI rat model. **a**-**g** Markers of myocardia injury cTnI, LDH and CK-MB and levels of MDA, SOD, GSH and T-AOC in WT and *Ucp1*^*−/−*^ rats with saline or ISO treatment after 3 days (*n* = 16/group, one-way ANOVA, †*P* < 0.05, ††*P* < 0.01 *versus* saline treatment in WT rats, *P < 0.05, **P < 0.01, ***P < 0.001 *versus* saline treatment of the same strain, ^#^*P* < 0.05, ^##^*P* < 0.01, ^###^*P* < 0.001 *versus* ISO treatment in WT rats). **h** Apoptosis was analyzed using in situ TUNEL fluorescence staining. The nuclei of TUNEL-positive (apoptotic) cells appeared green (magnification × 630, scan bar is 20 μm). **i** TEM analysis of mitochondria (black arrow) of LV apical segments (magnification × 25,000, scan bar is 1 μm)

Consistent with increased fibrosis, ISO treatment induced a significantly greater increase in serum cTnI levels in *Ucp1*^*−/−*^ rats than in WT littermates (52.43% *versus* 41.82%, *P* < 0.001, Fig. 3a). Additional serum markers of myocardial injury LDH and CK-MB (Fig. 3b, c) followed similar patterns, suggesting that UCP1 knockout increased the degree of myocardium injury induced by ISO.

Moreover, we detected the level of oxidant MDA, antioxidant SOD, GSH and T-AOC in serum, which reflect the degree of oxidative stress. The serum MDA levels in *Ucp1*^*−/−*^ rats was significantly increased compared with that of in WT littermates (1.94 folds *versus* 1.06 folds, *P* < 0.001, Fig. 3d), while the serum SOD levels in *Ucp1*^*−/−*^ rats was significantly decreased compared with that of in WT littermates (50.97% *versus* 44.10%, *P* < 0.05, Fig. 3e) induced by ISO. GSH and T-AOC followed similar patterns as well as SOD (Fig. 3f, g). UCP1 knockout increased ROS production and, in turn, caused progressive myocardial apoptosis (Fig. 3h).

Disrupted ultrastructure, such as swollen mitochondria with a loss of cristae and vacuolization were both appeared in WT and *Ucp1*^*−/−*^ rats after ISO treatment, while *Ucp1*^*−/−*^ rats presented worse mitochondria injury (Fig. 3i).

### UCP1 knockout exacerbated the myocardial PCr/ATP-ratio drop in ISO-induced AMI rat model

**Fig. 4:**
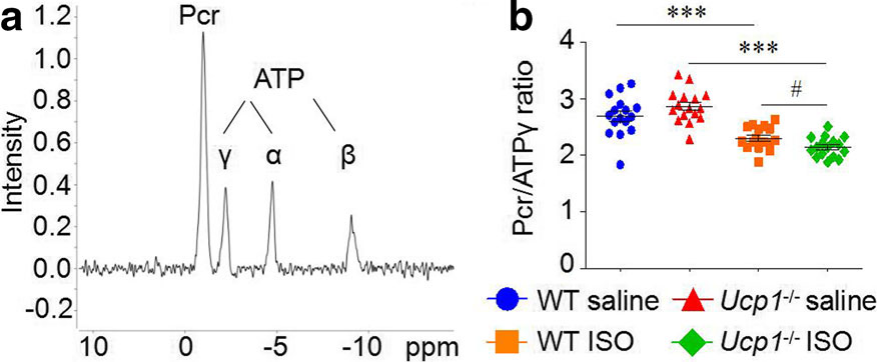
UCP1 knockout exacerbated the myocardial PCr/ATP-ratio drop in ISO-induced AMI rat model. **a** Typical cardiac localized ^31^P spectra obtained in vivo at 7.0 T in rats. **b** Myocardial PCr/ATPγ ratio in WT and *Ucp1*^*−/−*^ rats with saline or ISO treatment after 3 days (*n* = 16/group, one-way ANOVA, ***P < 0.001 *versus* saline treatment of the same strain, #*P* < 0.05 *versus* ISO treatment in WT rats)

We measured myocardial PCr and ATP content using MR-Spectroscopy, which reflected alterations of cardiac energy balance. Because of the difficulty of using spectroscopy to determine absolute quantities in vivo, we used the PCr/ATPγ ratio as a surrogate of energy balance. Our results demonstrated that the PCr/ATPγ ratio were comparable between WT and *Ucp1*^*−/−*^ rats treated with saline (2.87 ± 0.29 versus 2.70 ± 0.35), while the PCr/ATPγ ratio dropped more markedly (23.91% *versus* 13.78%, *P* < 0.05, Fig. 4) after ISO treatment in *Ucp1*^*−/−*^ rats compared with that of in WT littermates, indicated a defect in cardiac energy regulation in *Ucp1*^*−/−*^ rats heart.

### UCP1 knockout inhibited AMPK/mTOR/PPARα pathways in ISO-induced AMI rat model

**Fig. 5:**
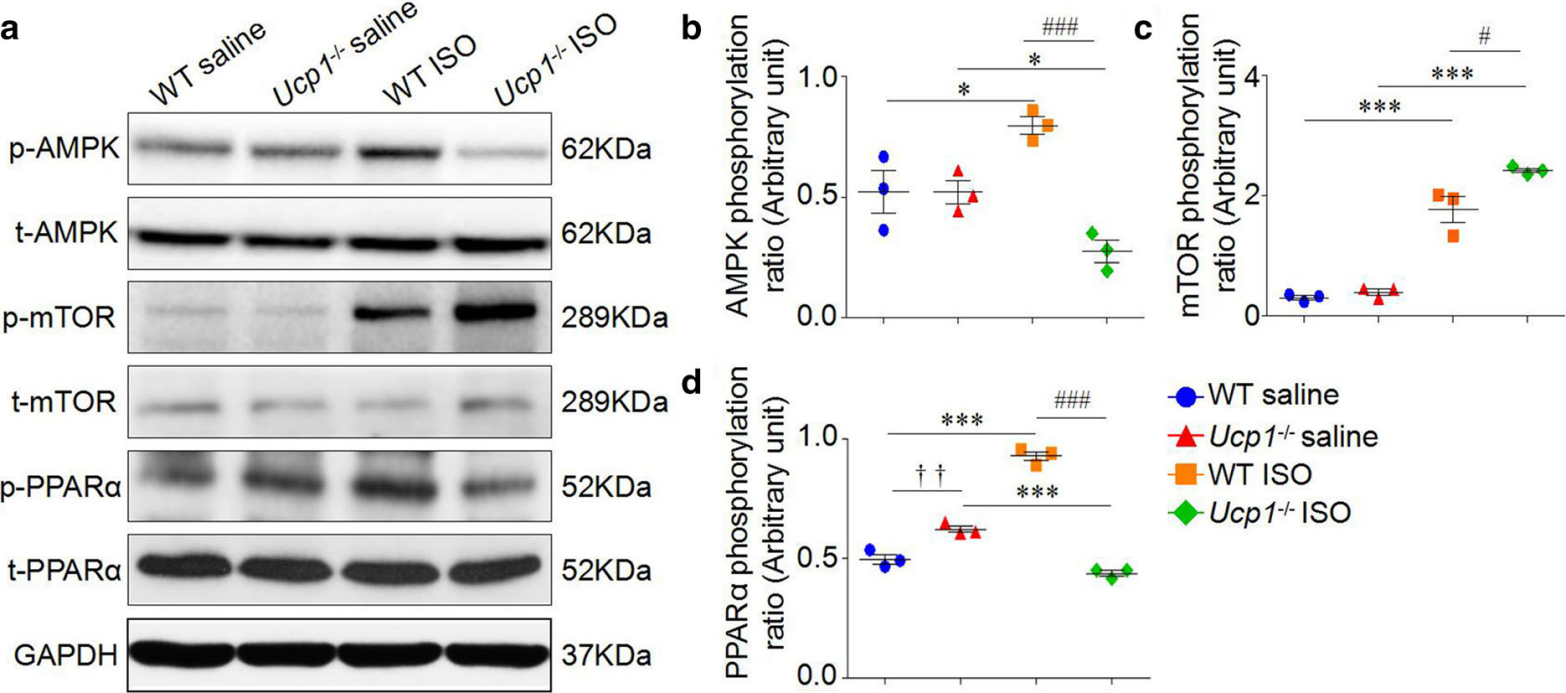
UCP1 knockout inhibited AMPK/mTOR/PPARα pathways in ISO-induced AMI rat model. **a** The phosphorylation level of AMPK, mTOR and PPARα were measured by western blot in the myocardium of WT and *Ucp1*^*−/−*^ rats with saline or ISO treatment after 3 days. **b**-**d** The quantitative analysis of the level of phosphorylated AMPK, mTOR and PPARα, using total AMPK, mTOR and PPARα for normalization (*n* = 3 independent experiments, one-way ANOVA, ††*P* < 0.01 *versus* saline treatment in WT rats, *P < 0.05, ***P < 0.001 *versus* saline treatment of the same strain,^#^*P* < 0.05,^###^*P* < 0.001 *versus* ISO treatment in WT rats)

*Ucp1* gene expression is mainly regulated by PPARα, which is a downstream signaling molecule of AMPK/mTOR pathways. AMPK is an energy sensor of heart and regulates myocardial metabolism. Therefore, we measured the phosphorylation level of AMPK, mTOR and PPARα in the heart tissue from WT and *Ucp1*^*−/−*^ rats with saline or ISO treatment after 3 days. We found that there was no significant difference in the levels of phosphor-AMPK and phosphor-mTOR in WT and *Ucp1*^*−/−*^ rats after saline treatment, while the level of phosphor-AMPK was significantly increased, and subsequently the level of phosphor-mTOR was decreased and phosphor-PPARα was increased in WT littermates after ISO treatment, whereas the activation of AMPK, inhibition of mTOR and activation of PPARα induced by ISO were significantly inhibited in *Ucp1*^*−/−*^ rats (Fig. 5).

## Discussion

**Fig. 6:**
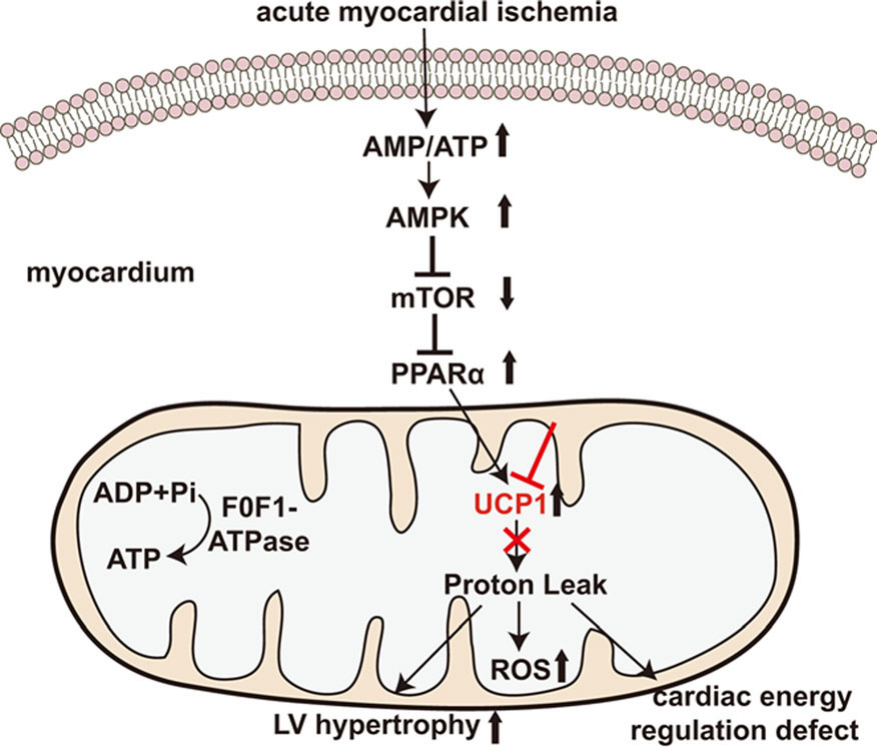
Schematic diagram showing the mechanism of UCP1 expression and its pathophysiological roles in ISO-induced AMI rat model

In the present study, we demonstrated that the expression of UCP1 was significantly increased in the heart tissue of ISO-induced AMI rat model. Knockout UCP1 expression aggravated adverse LV remodeling; increased myocardial injury, oxidative stress, myocardial apoptosis and mitochondria lysis; and exacerbated cardiac energy regulation defect induced by ISO. AMPK/mTOR/PPARα signaling pathway activation was involved in the upregulation of UCP1 in ISO-induced AMI rat model (Fig. 6).

The physiological function of UCP1 in BAT is mainly regulated by a balance between inhibition of proton transport by cytosolic guanine nucleotide diphosphate or triphosphate and activation of free fatty acids (Nicholls and Locke 1984). In normoxic heart, the UCP1 activity is almost inhibited because of the high ATP and low free fatty acid levels. During ischemia, oxidative phosphorylation is arrested resulting in decreased level of ATP, increased level of adenosine monophosphate (AMP) and the accumulation of ROS (Rohailla et al. 2014). High AMP/ATP ratio can activate AMPK, which is a key energy sensor of heart and regulates myocardial metabolism to maintain myocardium energy homeostasis (Yang et al. 2017; Zhang et al. 2019). Increased AMPK activity is an adaptation to ischemia and can subsequently inhibit the activity of mTOR, a central cell-growth regulator that integrates growth factor and nutrient signals. The AMPK/mTOR signaling pathway has been reported to have a cardioprotective effect on cardiac ischemic diseases (Li et al. 2017; Ma et al. 2020; Shi et al. 2017). For example, AMPK/mTOR signaling pathway was involved in the protection of melatonin against myocardial ischemia/reperfusion injury (Chen et al. 2018). AMPK associated activator, thyroid hormone, was shown to improve the mechanical performance of diabetic myocardium after infarction (Mourouzis et al. 2013). In this study, we demonstrated that the levels of phosphor-AMPK and phosphor-mTOR were comparable between WT and *Ucp1*^*−/−*^ rats after saline treatment, while the levels of phosphor-AMPK was significantly increased, and subsequently phosphor-mTOR was decreased in WT littermates after ISO treatment, whereas the activation of AMPK/mTOR pathways induced by ISO were significantly inhibited in *Ucp1*^*−/−*^ rats, suggesting that UCP1 knockout inhibited the cardioprotective activation of AMPK/mTOR pathways in ISO-induced AMI rat model. Furthermore, our results also showed that the degree of LV hypertrophy induced by ISO was greater in *Ucp1*^*−/−*^ rats than in WT littermates which may be due to inactivation of AMPK. It is reported that AMPK is also closely associated with the development of cardiac hypertrophy. The activation of AMPK has been shown to suppress myocardial cell protein synthesis and hypertrophic growth, thus inhibiting cardiac hypertrophy (Ma et al. 2016).

The *Ucp1* gene distal enhancer contains a complex nuclear receptor binding site which regulates the transcriptional activation of the *Ucp1* gene via PPAR agonists. The *Ucp1* gene is a target of dual regulation by PPARγ and PPARα in BAT. However, in already differential brown adipocytes PPARγ may not be essential for *Ucp1* gene expression (Nedergaard et al. 2005). *Ucp1* gene expression is induced mainly through PPARα in mature brown adipocytes, which is a downstream signaling molecule of AMPK/mTOR pathways (Villarroya et al. 2007). Our results indicated that the level of phosphor-PPARα was increased significantly in WT littermates after ISO treatment, and the activation of PPARα may induce the expression of UCP1 in the heart tissue of ISO-induced AMI rat model. Moreover, it is reported that during ischemia the level of inhibiting nucleotides (∑ATP + adenosine diphosohate (ADP)) was decreased meanwhile the level of UCP1 activators was increased (Hoerter et al. 2004). These above results may contribute to explain the upregulated expression of UCP1 in ISO-induced AMI rat model together. The ischemia-related injuries are the results of a combination of substrate and oxygen deprivation and ROS production (Opie and Sack 2002; Sadek et al. 2002). Most of ROS originate from mitochondrial, and their production increases with the decrease of the respiratory chain components, resulting in a high membrane potential. The upregulation of UCP1 would bring proton conductance, making oxidation rate (uncoupling) faster and membrane potential lower, consequently reduce ROS production by mitochondria(Sack 2006). Parameters linked to oxidative damage were estimated. ISO treatment induced a significantly greater increase in serum oxidant MDA levels, meanwhile a significantly greater decrease in serum antioxidant SOD, GSH and T-AOC levels in *Ucp1*^*−/−*^ rats than in WT littermates. Therefore, knockout UCP1 increases oxidative stress and the protective effect of UCP1 is related to the reduction of oxidative stress.

Alterations in cardiac energy balance may lead to various pathological features of the heart, which may not only be secondary to ventricular remodeling, but rather be an early feature of cardiac disease process (Holloway et al. 2012). The energy balance of mammalian heart refers to the dynamic homeostasis of PCr, ATP and related biochemical potentials in the myocardium. The PCr/ATP ratio will drop to lower values when cardiac energy metabolism is defective (Deschodt-Arsac et al. 2016). We detected the PCr/ATP ratio of myocardium and found that ISO treatment induced significantly drop in both WT and *Ucp1*^*−/−*^ rats, while PCr/ATP ratio of *Ucp1*^*−/−*^ rats dropped more markedly, indicating a defect in cardiac energy regulation in *Ucp1*^*−/−*^ rats heart.

Moreover, it is well known that UCP1 is expressed largely in BAT of mammals. Our results showed that BAT volume was significantly lower in *Ucp1*^*−/−*^ rats compared with that of WT littermates at baseline. Recent research indicates that BAT exerts a systemic cardioprotective effect (Thoonen et al. 2015). When activated, BAT would release several signaling molecules with endocrine properties (Stanford et al. 2013; Virtue et al. 2012) which target the heart. For example, fibroblast growth factor 21 (FGF21), one of the BAT synthetic adipokines, has been demonstrated cardiac antihypertrophic effects (Planavila et al. 2013) and cardioprotective effects on experimental MI mouse model (Liu et al. 2013). Therefore, it is possible that knockout UCP1 would decrease the cardioprotective adipokine levels secreted by BAT since the decrease of BAT volume.

In conclusion, the activation of AMPK/mTOR/PPARα signaling pathway was involved in the upregulation of UCP1 in the heart tissue of AMI of rats. UCP1 knockout aggravated ISO-induced AMI. Increasing UCP1 expression in heart tissue may be a cytoprotective therapeutic strategy for AMI.

## Supplementary Information

Below is the link to the electronic supplementary material.

Supplementary file1 (DOCX 199 kb)

## Data Availability

All data generated or analyzed during this study are included in this published article and its supplementary materials.
