# Supplementary material for: Uncoupling protein 1 knockout aggravates isoproterenol-induced acute myocardial ischemia via AMPK/mTOR/PPARα pathways in rats
Source: Transgenic Res. 2021 Oct 28;31(1):107–18. doi: 10.1007/s11248-021-00289-0 (PMC8821478; doi:10.1007/s11248-021-00289-0)
Supplement: Supplementary file 1 — Supplementary file1 (DOCX 199 kb) [file 11248_2021_289_MOESM1_ESM.docx]

**Transgenic Research**

**Uncoupling protein 1 knockout aggravates isoproterenol-induced acute myocardial ischemia via AMPK/mTOR/PPARα pathways in rats**

Daorong Hou^1,*^, Heling Fu^1,*^, Yuan Zheng^1^, Dan Lu^2^, Yuanwu Ma^2^, Yuan Yin^1^, Lianfeng Zhang^2^ and Dan Bao^1^

^1^Key laboratory of the model animal, Animal Core Facility of Nanjing Medical University, Nanjing, 211166, China.

^2^Key laboratory of Human Disease Comparative Medicine, NHFPC, Institute of Laboratory Animal Science, Chinese Academy of Medical Sciences & Comparative Medical Center, Peking Union Medical College, Beijing, 100021, China.

^*^ These authors contributed equally to this work.

**Corresponding author:** Dan Bao, PhD. and Lianfeng Zhang, PhD.

Key laboratory of the model animal, Animal Core Facility of Nanjing Medical University, 101 Longmian Avenue, Nanjing, 211166, China.

Key laboratory of Human Disease Comparative Medicine, NHFPC, Institute of Laboratory Animal Science, Chinese Academy of Medical Sciences & Comparative Medical Center, Peking Union Medical College, Beijing, 100021, China.

Tel.: +86 025-86867151 E-mail: [jndwbaodan@njmu.edu.cn](mailto:jndwbaodan@njmu.edu.cn) and [Zhanglf@cnilas.org](mailto:Zhanglf@cnilas.org)

**Supplementary Material**

**Supplemental Tables**

To determine whether UCP1 affects the cardiac geometry and function in ISO-induced acute myocardial injury, we generated *Ucp1* knockout rat using CRISPR/Cas9 system. We designed three pairs of synthesized oligonucleotides for gRNA targeting on the exon 1 of *Ucp1*. The gRNA sequences were shown in the followed Supplemental Table S1. The rat was genotyped by PCR and primers were shown in the followed Supplemental Table S2.

Table S1. Three pairs of gRNA targeting on *Ucp1*

|  | up sequence | down sequence |
| --- | --- | --- |
| *Ucp1* E1(1)-gRNA | TAGGGGTCAAGATCTTCTCAGC | AAACGCTGAGAAGATCTTGACC |
| *Ucp1* E1(2)-gRNA | TAGGTGATGATGTCTGCTAGGC | AAACGCCTAGCAGACATCATCA |
| *Ucp1* E1(3)-gRNA | TAGGACTTTGGCGGTGTCCAGC | AAACGCTGGACACCGCCAAAGT |

Table S2. PCR primers for genotyping of *Ucp1* knockout rats

|  | Sequence (5’→3’) |
| --- | --- |
| *Ucp1* forward | GCGATCCGGGCTTAAAGAG |
| *Ucp1* reverse | GTGCCCTCAAAGAATCAAATTAAGG |

To verify whether knockout of UCP1 expression could affect the cardiac geometry and function in ISO-induced acute myocardial injury, firstly, M-mode echocardiography was performed at 1, 3, 5 and 7 months of age on *Ucp1^-/-^* rats as well as WT littermates. The data was shown in the followed Supplemental Table S3. Furthermore, M-mode echocardiography was also performed on rats treated with saline and ISO intraperitoneal injection, respectively, in both of WT littermates and *Ucp1^-/-^* rats at 3 days and 1 month after ISO treatment. The data was shown in the followed Supplemental Table S4 and S5.

Table S3. Echocardiographic characteristics of rats at 1,3,5 and 7 months of age

|  | 1M | | 3M | | 5M | | 7M | |
| --- | --- | --- | --- | --- | --- | --- | --- | --- |
| Group | WT | *Ucp1^-/-^* | WT | *Ucp1^-/-^* | WT | *Ucp1^-/-^* | WT | *Ucp1^-/-^* |
| Number | n=17 | n=16 | n=18 | n=20 | n=15 | n=16 | n=15 | n=16 |
| LVEDD, mm | 6.26±0.65 | 6.16±0.64 | 7.71±1.22 | 8.10±1.13 | 7.80±0.89 | 8.26±0.84 | 8.44±0.62 | 8.98±1.21 |
| LVESD, mm | 3.40±0.77 | 3.42±0.58 | 4.39±1.26 | 4.79±1.13 | 4.49±0.91 | 5.16±0.82 | 5.27±0.60 | 5.78±1.16 |
| LVPWD, mm | 1.67±0.34 | 1.47±0.31 | 2.03±0.40 | 1.83±0.36 | 2.22±0.27 | 1.70±0.33 † † † | 2.25±0.34 | 1.74±0.28 † † † |
| LVPWS, mm | 1.99±0.42 | 1.93±0.62 | 2.24±0.26 | 1.98±0.30 † † † | 2.30±0.25 | 1.91±0.25 † † † | 2.31±0.40 | 1.82±0.26 † † † |
| LVAWD, mm | 1.33±0.19 | 1.31±0.17 | 1.64±0.30 | 1.57±0.34 | 1.70±0.35 | 1.53±0.31 | 1.69±0.31 | 1.47±0.38 |
| LVAWS, mm | 1.55±0.20 | 1.47±0.16 | 1.73±0.24 | 1.71±0.40 | 1.78±0.21 | 1.47±0.23 † † † | 1.71±0.23 | 1.45±0.40 † |
| LVEF, % | 75.37±9.96 | 74.31±7.92 | 72.99±9.67 | 69.73±9.14 | 71.56±8.73 | 65.81±5.90 † | 65.45±5.60 | 62.64±9.83 |
| LVFS, % | 45.96±9.12 | 44.61±7.44 | 44.22±8.44 | 41.41±7.87 | 42.77±7.19 | 37.84±4.49 † | 37.62±4.36 | 35.87±7.27 |

†*P*<0.05, † †*P*<0.01, † † †*P*<0.001 *versus* WT rats.

Table S4. Echocardiographic characteristics of rats at 3 days after ISO treatment

|  | saline | | ISO | |
| --- | --- | --- | --- | --- |
| Group | WT | *Ucp1^-/-^* | WT | *Ucp1^-/-^* |
| Number | n=20 | n=20 | n=17 | n=18 |
| LVEDD, mm | 7.03±0.58 | 7.08±0.44 | 6.01±0.65*** | 5.47±0.46*** # |
| LVESD, mm | 4.22±0.55 | 4.22±0.46 | 3.05±0.48*** | 2.48±0.69*** # |
| LVPWD, mm | 1.60±0.24 | 1.51±0.25 | 2.11±0.35*** | 2.27±0.48*** |
| LVPWS, mm | 2.55±0.26 | 2.48±0.46 | 3.09±0.36*** | 3.35±0.57*** |
| LVAWD, mm | 1.36±0.16 | 1.34±0.17 | 1.75±0.18*** | 2.08±0.26*** ### |
| LVAWS, mm | 2.22±0.30 | 2.10±0.32 | 2.71±0.28*** | 3.27±0.46*** ### |
| LVEF, % | 69.14±5.41 | 69.54±5.63 | 79.47±5.38*** | 85.46±8.35*** # |
| LVFS, % | 40.13±4.54 | 40.51±4.78 | 49.33±5.39*** | 56.58±8.9*** # |

**P*<0.05, ***P*<0.01, ****P*<0.001 *versus* saline treatment of the same strain at 3 days;

#*P*<0.05, ##*P*<0.01, ###*P*<0.001 *versus* ISO treatment in WT rats.

Table S5. Echocardiographic characteristics of rats at 1 month after ISO treatment

|  | saline | | ISO | |
| --- | --- | --- | --- | --- |
| Group | WT | *Ucp1^-/-^* | WT | *Ucp1^-/-^* |
| Number | n=18 | n=20 | n=5 | n=5 |
| LVEDD, mm | 7.71±1.22 | 8.10±1.13 | 8.33±0.64 | 7.90±0.45 |
| LVESD, mm | 4.39±1.26 | 4.79±1.13 | 5.78±0.48* | 5.58±0.45 |
| LVPWD, mm | 2.03±0.40 | 1.83±0.36 | 1.62±0.24* | 1.85±0.29 |
| LVPWS, mm | 2.24±0.26 | 1.98±0.30 † † † | 2.43±0.26 | 2.37±0.28* |
| LVAWD, mm | 1.64±0.30 | 1.57±0.34 | 1.54±0.05 | 1.48±0.11 |
| LVAWS, mm | 1.73±0.24 | 1.71±0.40 | 2.37±0.04*** | 2.04±0.25 # |
| LVEF, % | 72.99±9.67 | 69.73±9.14 | 58.54±3.10** | 53.78±2.48*** # |
| LVFS, % | 44.22±8.44 | 41.41±7.87 | 32.51±2.25** | 28.94±1.98** # |

† † †*P*<0.001 *versus* saline treatment in WT rats;

**P*<0.05, ***P*<0.01, ****P*<0.001 *versus* saline treatment of the same strain at 3 days;

#*P*<0.05 *versus* ISO treatment in WT rats.

**Supplemental Figures**

Figure S1. ECG analysis for rat models.


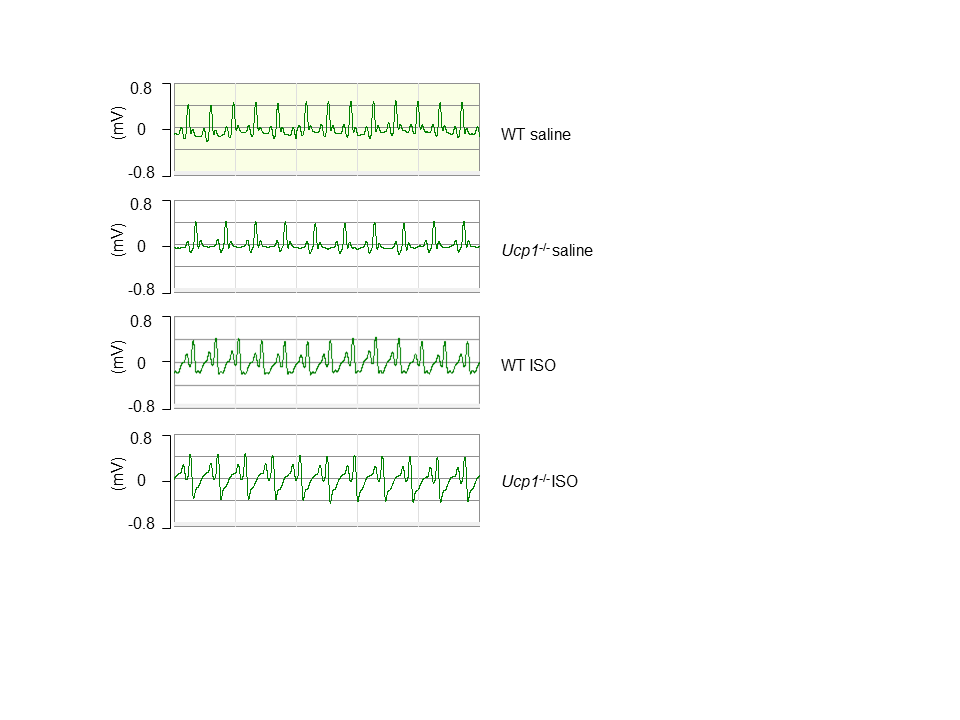


At 3 days after saline or ISO administration, WT-saline, *Ucp1^-/-^*-saline, WT-ISO and *Ucp1^-/-^*-ISO rats (n=20/group) were anesthetized with 1.5% isoflurane and placed in supine position. The ECG electrodes were fixed on both upper and right lower limbs of rats. The standard II-lead ECG was recorded using BIOPAC MP150 physiological signal acquisition system. The S-T segment alteration and/or T wave inversion of ECG indicated the successful establishment of ISO-induced AMI models.

Figure S2. Cumulative percent mortality for rat models.


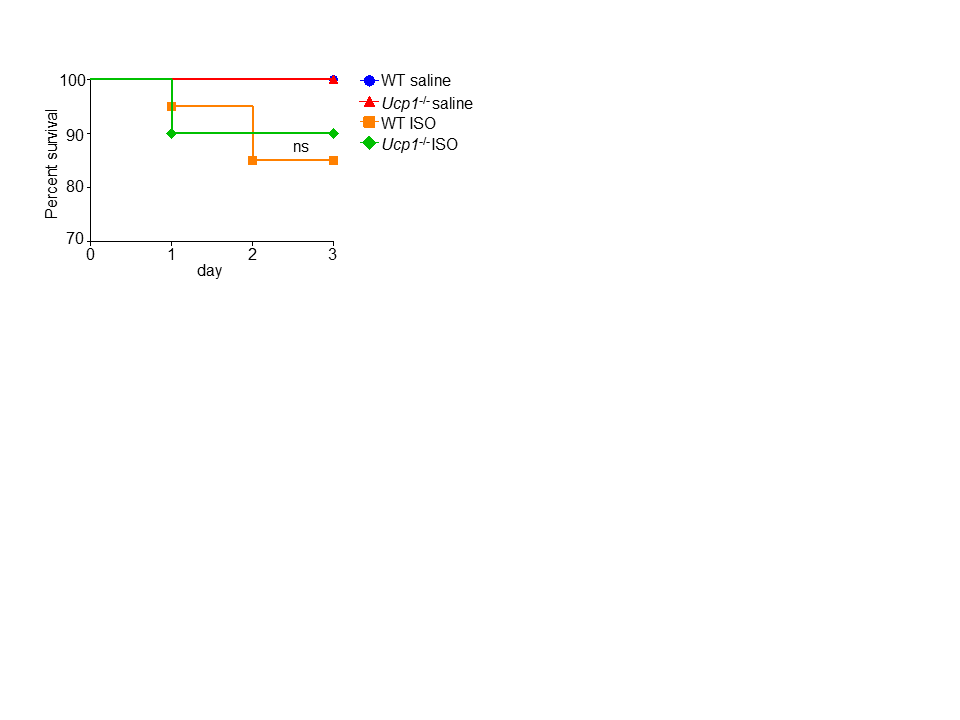


WT-saline, *Ucp1^-/-^*-saline, WT-ISO and *Ucp1^-/-^*-ISO rats (n=20/group) were calculated every day until the third day after ISO treatment (no significant difference *versus* ISO group).

Figure S3. LVEF of rats at 1 month after ISO treatment.


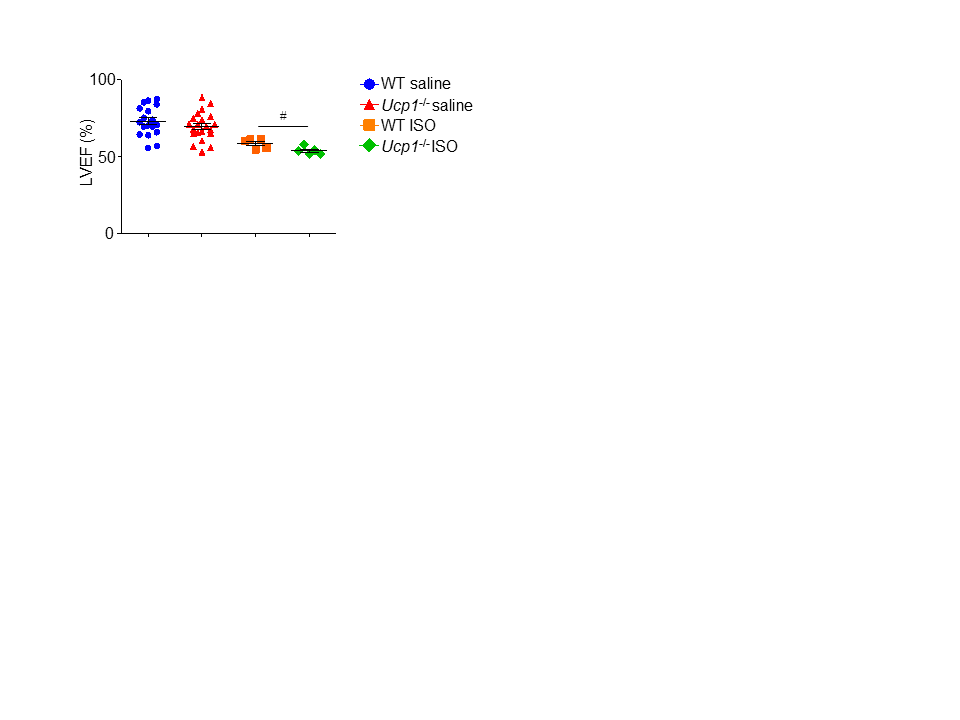


WT-saline (n=18), *Ucp1^-/-^*-saline (n=20), WT-ISO (n=5) and *Ucp1^-/-^*-ISO rats (n=5) were performed with M-mode echocardiography at 1 month after ISO treatment.

#*P*<0.05 *versus* ISO treatment in WT rats.
